# MicroRNA-454 may function as an oncogene via targeting AKT in triple negative breast cancer

**DOI:** 10.1186/s40709-017-0067-x

**Published:** 2017-08-03

**Authors:** Qun Li, Jia Liu, Xianying Meng, Renzhu Pang, Jie Li

**Affiliations:** The First Hospital, Jilin University, Changchun, Jilin China

**Keywords:** miR-454, Triple negative breast cancer, Apoptosis, AKT

## Abstract

**Background:**

Altered microRNAs expression mediates tumor development and progression in many type cancers including triple negative breast cancer (TNBC). Here we detected the effect of miR-454 on cell proliferation, migration and invasion of triple negative breast cancer cells.

**Results:**

miR-454 promoted the proliferation of TNBC, and enhanced migration and invasion in TNBC cells. Meanwhile, miR-454 improved the survival of TNBC cells after ironizing radiation. miR-454 inhibited radiation-induced apoptosis in TNBC cells by regulation of caspase 3/7 and Bcl-2 expression. Furthermore, PTEN and pAKT levels in TNBC cells were changed after overexpression of miR-454.

**Conclusions:**

miR-454 played an essential role in tumor development and progression in TNBC, and might be used as a potential biomarker to predict radiotherapy response and prognosis in TNBC.

## Background

Breast cancer remained the most commonly diagnosed cancer in women and the second leading cause of cancer death among women [1]. In the past decade, early detection and screening and improvement in medical care and treatments decreased dramatically the mortality of patient with breast cancer [2, 3].

Triple-negative breast cancer (TNBC) is a subtype of breast cancer characterized as negative for estrogen receptors, progesterone receptors, and HER2 [4]. Studies have demonstrated that TNBC is an aggressive disease with unique molecular profile [5]. Brain and lungs are the most common metastasis sites of TNBC [6]. There are no targeted therapies for TNBC. Therefore, it is important to discovery of new molecular targets to treat patients with TNBC [7, 8].

It is well known that microRNAs (miRNAs) play critical roles in regulation of gene expression [9]. Studies have demonstrated that miRNAs can behave as both oncogenes and tumor suppressor genes [10]. MiRNAs regulate many biological processes, such as cell proliferation, apoptosis and cell cycle. Several studies have shown that miR-10b improves cell migration and invasion by targeting HOXD10 [11, 12]. Huang et al. has reported that miR-21 is associated with breast cancer cell invasion and regulated epithelial-to-mesenchymal transition (EMT) [13]. Han et al. [14] has shown that miR-21 regulates breast cancer stem cell-like cells.

Zhang et al. has reported that miR-155 improves breast cancer cell proliferation by regulation of p53 [15]. miR-125b is downregulated in breast cancer and correlates with metastasis and HER2 expression [16]. Recent studies indicate that miRNAs are associated with prognosis in breast cancer [17] while miR-454 plays critical roles in tumor development in many organs [18, 19]. Zhu et al. found that miR-454 promoted proliferation of non-small cell lung cancer cells by targeting PTEN [20]. The oncogene role of miR-454 is also found in uveal melanoma [18]. Yu et al. reported that miR-454 enhanced hepatocellular carcinoma cell proliferation, invasion and epithelial mesenchymal transition [21]. Liu et al. [19] and Liang et al. [22] showed that miR-454 regulated cell proliferation in colon cancer by different signaling pathways.

In this study, we examined the function of miR-454 on cell proliferation with or without radiation, invasion and migration using triple-negative breast cancer cell lines.

## Methods

### Cell lines and cell culture

Two human triple-negative breast cancer cell lines, MDA-MB-231 and MDA-MB-468 (ATCC, USA), were used in all experiments in our study in order to ensure all data are not cell-line restricted. These tumor cells were cultured in Dulbecco’s modified Eagle’s medium (DMEM) (Invitrogen, USA) supplemented with 100 µg ml^−1^ streptomycin, 100 U ml^−1^ penicillin and 10% fetal bovine serum (Invitrogen, USA) at 37 °C in a humidified incubator with 5% CO_2_. When all cells reach to 80% confluence, they were harvested and used in our experiments.

### Cell transfection and miRNA quantification

Lipofectamine 2000 (Invitrogen, USA) was used to transfer miR-454 mimics or anti-miR-454 inhibitor (Invitrogen, USA) into TNBC cells. A random sequence miRNA mimic molecule was used as a negative control (mirVana™miRNA mimic, Ambion, USA). Then, miR-454 expression level was examined by real time PCR after transfection. The total RNA was extracted from the transfected TNBC cells; TaqMan microRNA reverse transcription kit (ThermoFisher, USA) was used to synthesize cDNA according the manufacturer’s instructions. GAPDH was used as the endogenous reference gene.

### Cell proliferation assay

WST-1 assay (Roche, USA) was performed to analyze the function of miR-454 on TNBC cell proliferation. Briefly, the transfected TNBC cells were seeded into 96-well plates at the density of 2 × 10^4^ cells well^−1^. The cells were cultured overnight at 37 °C in a humidified incubator with 5% CO_2_. Then, the TNBC cells were exposed to different doses of ionizing radiation. Then WST-1 reagent was added to each well and incubated for 1 h at 37 °C every 24 h. Afterwards, the absorbance was measured at 490 nm. All experiments were performed in triplicates.

### Migration and invasion assays

The Promega migration and invasion assays were performed to evaluate migration and invasion of TNBC cell following the manufacturer’s instructions (Promega Corporation, USA). Briefly, transfected TNBC cells were placed on the upper transwell chamber either present or absent matrigel in DMEM. DMEM supplemented with 5% FBS was added to the lower chamber. The cells were cultured at 37 °C for 18 h. Then Diff-Quik stain (Invitrogen, USA) was performed after the non-invaded cells were removed by cotton swabs. The migration and invasion was calculated and showed as a ratio of invaded cells over cells normalized on second day of growth curve.

### Western blot assay

The TNBC cells were harvested and gently washed with cold phosphate buffered saline. The cells were lysed by adding ice-cold lysis buffer (50 mM Tris–HCl, pH 7.5, 0.1% SDS, 150 mM NaCl, 0.5% deoxycholate, 1% NP-40, and 1× protease inhibitors). The mixture was boiled at 100 °C for 5 min. The same amount of protein lysates were loaded to the SDS-PAGE gel and transferred to PVDF membranes (Sigma, USA). The PVDF membranes were incubated in blocking buffer at room temperature for 1 h. Primary antibodies (Bcl-2, Bax, AKT and pAKT; Cell Signaling Technology, USA) were added in TBS-T buffer and incubated at 4 °C overnight, followed by secondary antibody incubation at room temperature for 1 h. The signals were examined with the EasySeeWeatern Blot Kit (Transgen, Shanghai).

### Apoptosis activity assay

To examine the apoptosis activity of TNBC cells with altered miR-454 expression, the TNBC cells were grown in 24-well plates at density of 1 × 10^5^ well^−1^. Then the TNBC cells were exposed to different doses of ionizing radiation. The TNBC cells were continued to culture for 24 h, the caspase-Glo3/7 assay kit (Promega, Madison, USA) was used to examine apoptosis activity by measure the caspase 3/7 activity following the manufacturer’s protocol. Briefly, caspase-Glo reagent was added to the TNBC cells, and incubated at room temperature in a dark place with gentle shaking for 8 h. The luminescence value was measured using 1-min lag time and 0.5 s well^−1^ read time. All experiments were carried out in triplicates.

### Statistical analysis

SPSS software (version 11.0, IBM, USA) was used for statistical analyses using Student’s t test. Differences are considered statistically significant if *p* < 0.05.

## Results

### miR-454 increased the proliferation of TNBC cells and improved the survival at ionizing radiation

**Fig. 1:**
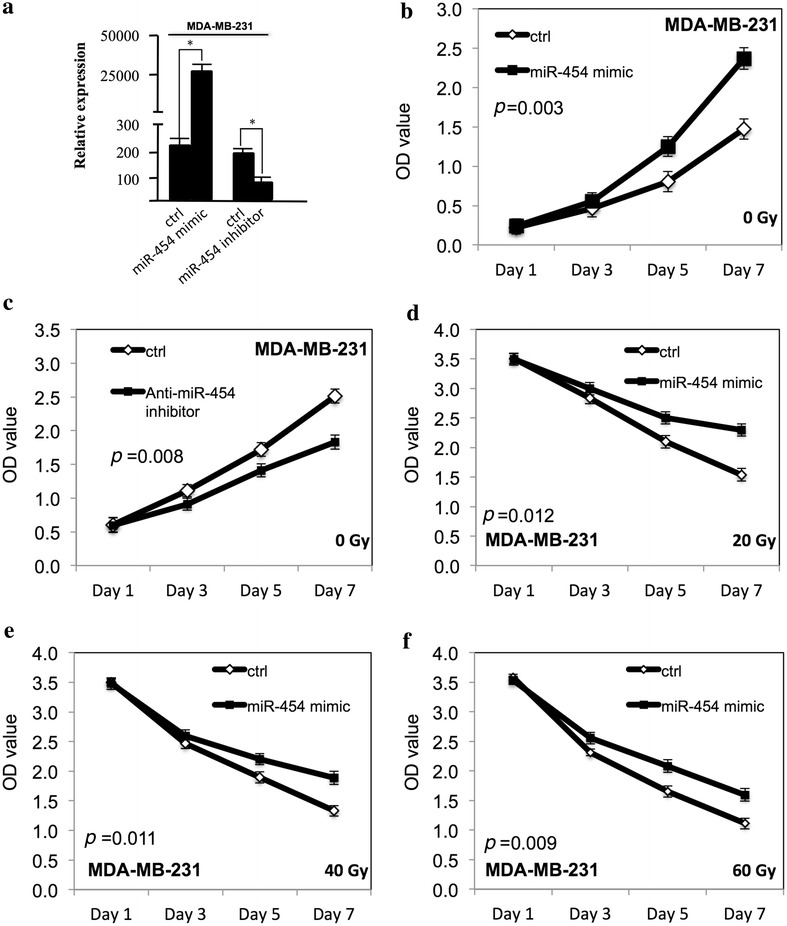
miR-454 increased the proliferation of MDA-MB-231 cells and improved the survival at ionizing radiation. **a** The miR-454 expression level was determined by qRT-PCR in MDA-MB-231 cells transfected with either miR-454 mimic or anti-miR-454 inhibitor. **b** The proliferation of MDA-MB-231 cells after transfection of miR-454 mimic. **c** The proliferation of MDA-MB-231 cells after transfection with anti-miR-454 inhibitor. **d** The proliferation of MDA-MB-231 cells after miR-454 overexpression at 20 Gy. **e** The proliferation of MDA-MB-231 cells after miR-454 overexpression at 40 Gy. **f** The proliferation of MDA-MB-231 cells after miR-454 overexpression at 60 Gy. All experiments were performed in triplicate

**Fig. 2:**
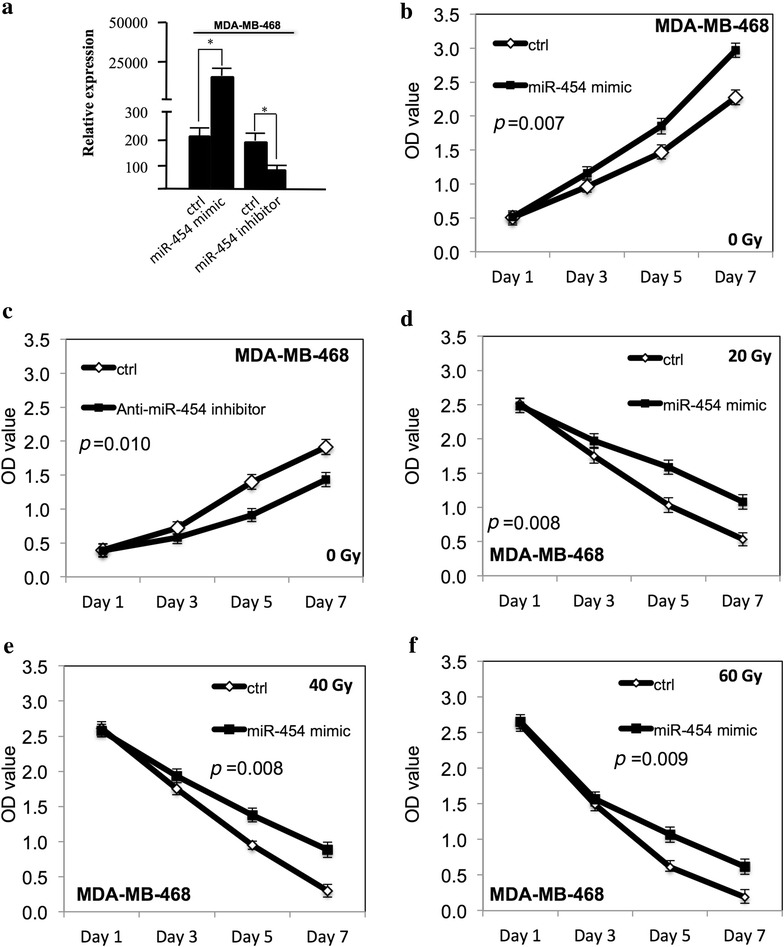
miR-454 increased the proliferation of MDA-MB-468 cells and improved the survival at ionizing radiation. **a** The miR-454 expression level was determined by qRT-PCR in MDA-MB-468 cells transfected with either miR-454 mimic or anti-miR-454 inhibitor. **b** The proliferation of MDA-MB-468 cells after transfection of miR-454 mimic. **c** The proliferation of MDA-MB-468 cells after transfection with anti-miR-454 inhibitor. **d** The proliferation of MDA-MB-468 cells after miR-454 overexpression at 20 Gy. **e** The proliferation of MDA-MB-468 cells after miR-454 overexpression at 40 Gy. **f** The proliferation of MDA-MB-468 cells after miR-454 overexpression at 60 Gy. All experiments were performed in triplicate

To examine the function of miR-454 on proliferation of TNBC cells, miR-454 was transfected to MDA-MB-231 and MDA-MB-468 TNBC cells with Lipofectamine 2000. WST-1 assay was performed to examine the proliferation. As shown in Figs. 1a and 2a, the miR-454 expression level was significantly increased in both MDA-MB-231 and MDA-MB-468 cells after overexpression of miR-454 mimic, and the miR-454 expression level was inhibited after transfection of anti-miR-454 inhibitor. miR-454 promoted the proliferation of MDA-MB-231 (Fig. 1b) and MDA-MB-468 cells (Fig. 2b) (*p* < 0.05). When the miR-454 level was inhibited by anti-miR-454 inhibitor, the proliferation was decreased in MDA-MB-231 (Fig. 1c) and MDA-MB-468 cells (Fig. 2c) (*p* < 0.05). When the TNBC cells were exposed to ionizing radiation, miR-454 improved the survival of MDA-MB-231 (Fig. 1d–f) and MDA-MB-468 cells (Fig. 2d–f) (*p* < 0.05) at dose dependent manner.

### miR-454 decreased radiation-induced apoptosis in TNBC cells

**Fig. 3:**
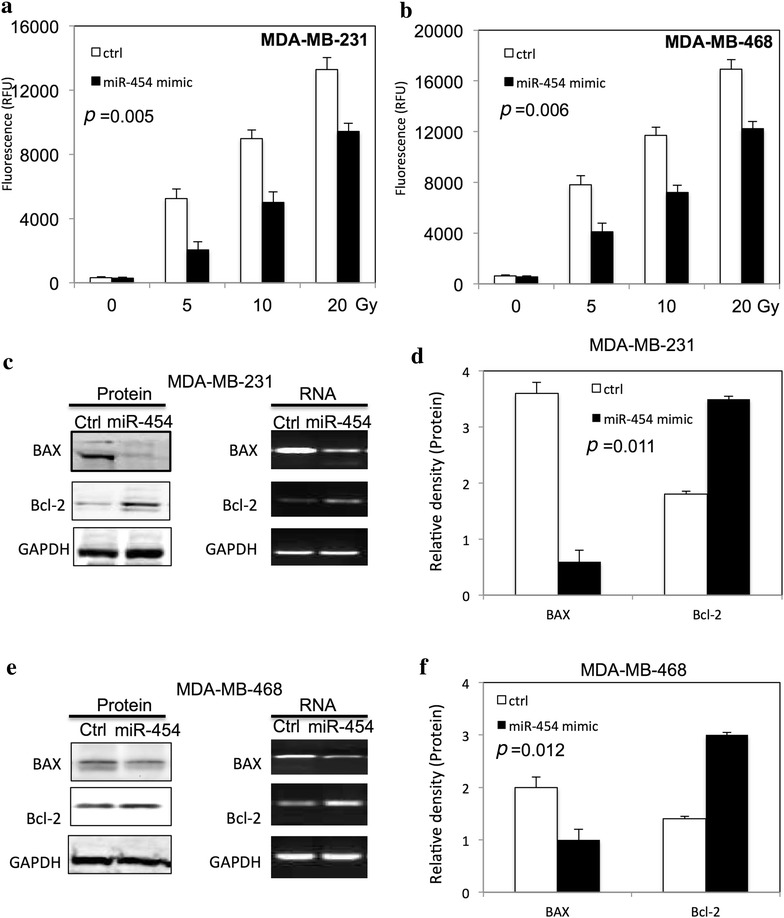
miR-454 inhibits radiation-induced apoptosis in TNBC cells. **a** The caspase 3/7 activity of MDA-MB-231 cells transfected with miR-454 mimic after different doses of ionizing radiation. **b** The caspase 3/7 activity in MDA-MB-468 cells transfected with miR-454 mimic after different doses of ionizing radiation. **c**, **d** The apoptotic proteins and RNA levels in MDA-MB-231 cells after transfection of miR-454 mimic. **e**, **f** The apoptotic proteins and RNA levels in MDA-MB-468 cells after transfection of miR-454 mimic. All experiments were performed in triplicate

**Fig. 4:**
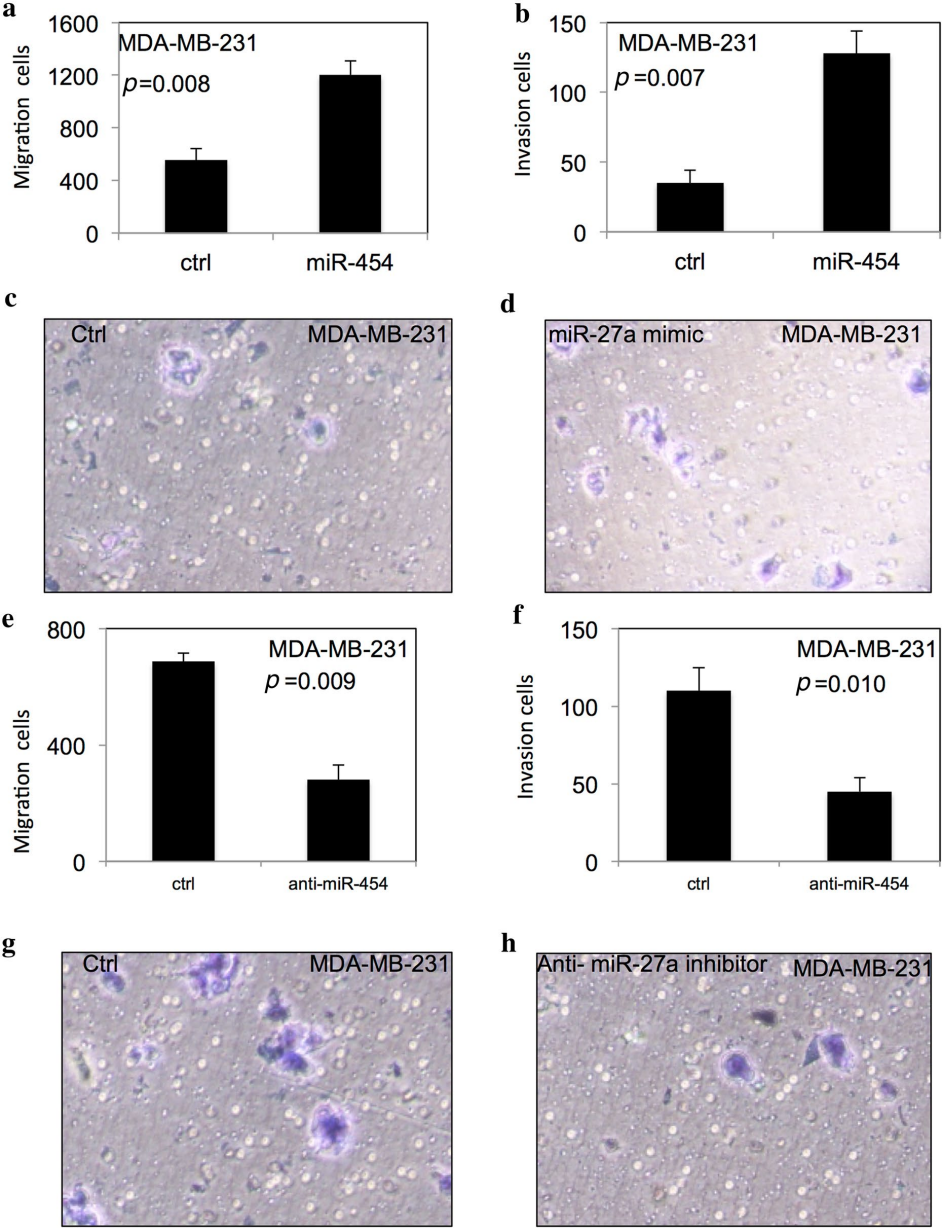
miR-454 improved migration and invasion of TNBC cells. miR-454 improved migration and invasion of MDA-MB-231 cells (**a**–**d**). The migration and invasion of MDA-MB-231 cells were decreased after downregulation of miR-454 (**e**–**h**). The migration and invasion of MDA-MB-468 cells were decreased after downregulation of miR-454 (**e**–**h**). All experiments were performed in triplicate

To determine the function of miR-454 on radiation-induced apoptosis in TNBC cells, the caspase-Glo 3/7 assay kit was used by measuring caspase 3/7 activity, and apoptotic related protein was examined by western blot. As shown in Fig. 3, miR-454 inhibited the caspase 3/7 activity in both MDA-MB-231 (Fig. 3a) and MDA-MB-468 (Fig. 3b) cells at dose dependent manner. The expression of BAX and Bcl-2 was altered in MDA-MB-231 (Fig. 4c, d) and MDA-MB-468 (Fig. 4e, f) after upregulation of miR-454.

### miR-454 improved migration and invasion of TNBC cells

**Fig. 5:**
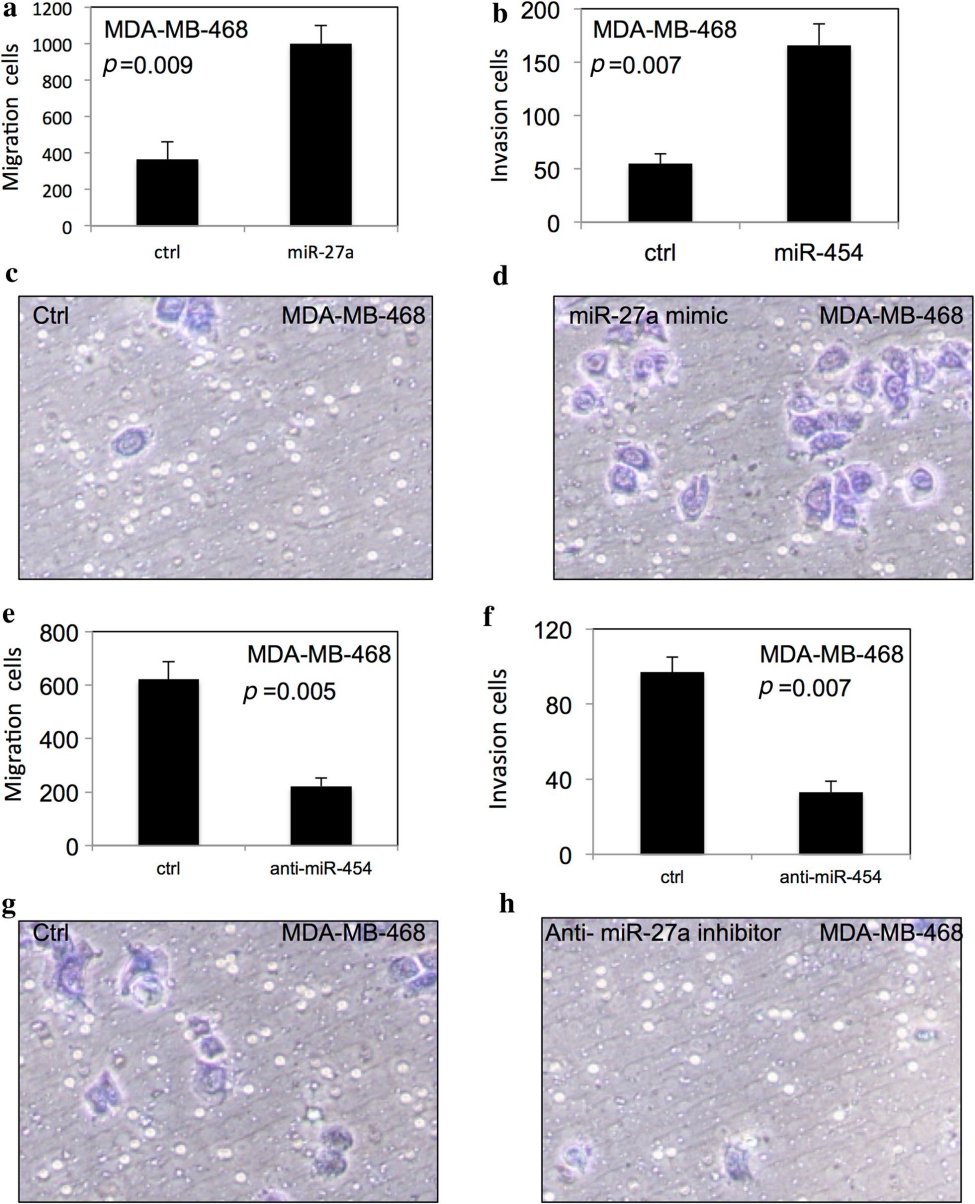
miR-454 improved migration and invasion of TNBC cells. miR-454 improved migration and invasion in MDA-MB-468 cells (**a**–**h**). All experiments were performed in triplicate

The migration and matrigel invasion assays were used to examine the effect of miR-454 on migration and invasion of TNBC cells using BD transwell. As shown in Fig. 4, the migration and invasion of MDA-MB-231 (Fig. 4a–d) and MDA-MB-468 (Fig. 5a–d) were significantly increased after overexpression of miR-454. In contrast, downregulation of miR-454 significantly decreased the migration and invasion of MDA-MB-231 (Fig. 4e–h) and MDA-MB-468 cells (Fig. 5e–h) (*p* < 0.05).

### miR-454 inhibited PTEN and activated pAKT in TNBC cells

**Fig. 6:**
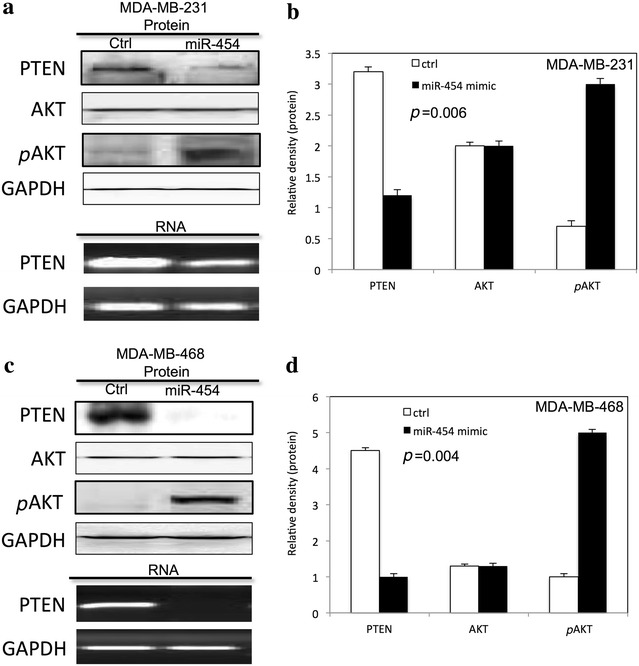
miR-454 activated pAKT in TNBC cells. **a** The expression of, pAKT, AKT and PTEN in MDA-MB-231 cells after overexpression of miR-454. **b** The relative expression level of proteins on MDA-MB-231 cells after overexpression of miR-454 by densitometric analysis. **c** The expression of pAKT, AKT and PTEN in MDA-MB-468 cells after overexpression of miR-454. **d** The relative expression level of proteins on MDA-MB-468 cells after overexpression of miR-454 by densitometric analysis. All experiments were performed in triplicate

The expression of pAKT, total AKT, and PTEN was detected in both MDA-MB-231 and MDA-MB-468 cells after miR-454 overexpression. We found that miR-454 decreased the PTEN expression level and increased the expression of pAKT in both MDA-MB-231 (Fig. 6a, b) and MDA-MB-468 (Fig. 6c, d) cells.

## Discussion

Recent studies have shown that miR-454 is highly expressed in tissue from patients with TNBC [23]. These findings indicate that miR-454 expression level might be positively correlated with worse clinical outcome. Therefore, miR-454 might act as a potential predictor of prognosis in TNBC. Recently, a tissue microarray study showed that miR-454 is associated with poor prognosis in patients with TNBC [23]. In our study, we found that miR-454 improved the proliferation of TNBC cells. Radiotherapy is one of highly effective adjuvant therapies in many types of cancer including breast cancer after surgery [24]. TNBC is a complex disease with features such as rapid growth and local recurrence. It has been reported that radiotherapy can decrease the locoregional recurrence in patients at early stage after surgery [25]. In the present study, we showed that miR-454 improved the survival of TNBC cells after ionizing radiation. Meanwhile, miR-454 inhibited the radiation-induced apoptosis of TNBC cells. Moreover, we showed that overexpression of miR-454 increased migration and invasion in TNBC cells. However, downregulation of miR-454 inhibited the migration and invasion in TNBC cells. It has been demonstrated that TNBC is more likely to recur and has poor prognosis [26]. Metastasis is one of important features for the recurrence of TNBC. Therefore, our study showed that miR-454 play critical roles in TNBC progression.

The PI3K/AKT pathway involves many biological processes and regulates the downstream responses including cell proliferation, apoptosis and cell cycle. It has been reported that PI3K/AKT is activated in TNBC due to PTEN mutation [27]. PTEN, known as the second most frequently mutated tumor suppressor gene in human cancer, plays important roles in proliferation, apoptosis and cell cycle in many type tumor cells. Recent studies have demonstrated that PI3K/AKT pathway is the key pathway by which PTEN displays antioncogenic functions [28]. Our results showed that PTEN expression level was downregulated, and pAKT expression level was increased in TNBC cells after overexpression of miR-454. These results indicated miR-454 mediated proliferation of TNBC cells by targeting PI3K/AKT signaling pathway. Further studies are needed to demonstrate the molecular mechanism.

## Conclusions

MiR-454 might act as an oncogene to regulate tumorigenesis and malignant progression of TNBC. Our study suggests miR-454 might be used as a potential biomarker to predict the radiotherapy response and prognosis in TNBC.
